# Pre-existing cell populations with cytotoxic activity against SARS-CoV-2 in people with HIV and normal CD4/CD8 ratio previously unexposed to the virus

**DOI:** 10.3389/fimmu.2024.1362621

**Published:** 2024-05-15

**Authors:** Guiomar Casado-Fernández, Juan Cantón, Laura Nasarre, Fernando Ramos-Martín, Mario Manzanares, Clara Sánchez-Menéndez, Daniel Fuertes, Elena Mateos, María Aranzazu Murciano-Antón, Mayte Pérez-Olmeda, Miguel Cervero, Montserrat Torres, Rafael Rodríguez-Rosado, Mayte Coiras

**Affiliations:** ^1^ Immunopathology and Viral Reservoir Unit, National Center of Microbiology, Instituto de Salud Carlos III, Majadahonda, Madrid, Spain; ^2^ PhD Program in Health Sciences, Faculty of Sciences, Universidad de Alcalá, Alcalá de Henares, Spain; ^3^ Internal Medicine Service, Hospital Universitario Severo Ochoa, Leganés, Madrid, Spain; ^4^ PhD Program in Biomedical Sciences and Public Health, Universidad Nacional de Educación a Distancia (UNED), Madrid, Spain; ^5^ Hematology and Hemotherapy Service, Instituto Ramón y Cajal de Investigación Sanitaria (IRYCIS), Hospital Universitario Ramón y Cajal, Madrid, Spain; ^6^ School of Telecommunications Engineering, Universidad Politécnica de Madrid, Madrid, Spain; ^7^ Biomedical Research Center Network in Infectious Diseases [Centro de Investigación Biomédica en Red Enfermedades Infecciosas (CIBERINFEC)], Instituto de Salud Carlos III, Majadahonda, Madrid, Spain; ^8^ Family Medicine, Centro de Salud Doctor Pedro Laín Entralgo, Alcorcón, Madrid, Spain; ^9^ International PhD School, Universidad Rey Juan Carlos, Alcorcón, Madrid, Spain; ^10^ Serology Service, Instituto de Salud Carlos III, Madrid, Spain; ^11^ School of Medicine, Universidad Alfonso X El Sabio, Madrid, Spain

**Keywords:** people with HIV, COVID-19 vaccination, Tγδ cells, cellular immune response, humoral immune response

## Abstract

**Introduction:**

HIV-1 infection may produce a detrimental effect on the immune response. Early start of antiretroviral therapy (ART) is recommended to preserve the integrity of the immune system. In fact, people with HIV (PWH) and normal CD4/CD8 ratio appear not to be more susceptible to severe forms of COVID-19 than the general population and they usually present a good seroconversion rate in response to vaccination against SARS-CoV-2. However, few studies have fully characterized the development of cytotoxic immune populations in response to COVID-19 vaccination in these individuals.

**Methods:**

In this study, we recruited PWH with median time of HIV-1 infection of 6 years, median CD4/CD8 ratio of 1.0, good adherence to ART, persistently undetectable viral load, and negative serology against SARS-CoV-2, who then received the complete vaccination schedule against COVID-19. Blood samples were taken before vaccination against COVID-19 and one month after receiving the complete vaccination schedule.

**Results:**

PWH produced high levels of IgG against SARS-CoV-2 in response to vaccination that were comparable to healthy donors, with a significantly higher neutralization capacity. Interestingly, the cytotoxic activity of PBMCs from PWH against SARS-CoV-2-infected cells was higher than healthy donors before receiving the vaccination schedule, pointing out the pre-existence of activated cell populations with likely unspecific antiviral activity. The characterization of these cytotoxic cell populations revealed high levels of Tgd cells with degranulation capacity against SARS-CoV-2-infected cells. In response to vaccination, the degranulation capacity of CD8+ T cells also increased in PWH but not in healthy donors.

**Discussion:**

The full vaccination schedule against COVID-19 did not modify the ability to respond against HIV-1-infected cells in PWH and these individuals did not show more susceptibility to breakthrough infection with SARS-CoV-2 than healthy donors after 12 months of follow-up. These results revealed the development of protective cell populations with broad-spectrum antiviral activity in PWH with normal CD4/CD8 ratio and confirmed the importance of early ART and treatment adherence to avoid immune dysfunctions.

## Introduction

Infection by the human immunodeficiency virus type 1 (HIV-1) may cause a severe impairment of the immune response (1). The rapid initiation of the antiretroviral therapy (ART) and continuous adherence to it may protect CD4+ T cells from HIV-1 infection and elimination, stabilizing the CD4/CD8 ratio (2). Therefore, among other determinations such as quantitative measurements of viral load (3), the CD4/CD8 ratio may be used as a quantitative outcome of both HIV-1 pathogenesis and disease progression and to determine the integrity of the immune response in people with HIV (PWH). CD4/CD8 below 0.4 has been associated with immune senescence and higher risk of morbimortality in aviremic PWH (4), while PWH with CD4/CD8 ratio greater than 1.0 show a healthy immune system that may respond efficiently to other infectious agents and to vaccination. In fact, vaccination is recommended in PWH with well-controlled HIV-1 infection and CD4 counts above 200 cells/µl (5), including vaccines against Influenza, Hepatitis A and B virus, pneumococcus, or SARS-CoV-2 (6, 7). CD4/CD8 is also considered a prognostic risk factor for other infectious diseases such as Coronavirus disease 2019 (COVID-19) (8–10) and high levels of CD4+ T cells have been associated with an adequate seroconversion in PWH after vaccination against COVID-19, while PWH with low CD4 counts may not respond adequately even after receiving the complete vaccination schedule (11). However, there is still not consensus if PWH have an increased risk of developing severe COVID-19 or not compared to healthy individuals or if they can response adequately to COVID-19 vaccination (12, 13), mostly due to the different progression of HIV-1 infection in each individual, the presence of comorbidities, and other social determinants of health (14).

The available data suggest that COVID-19 vaccines are safe in PWH, with the same incidence rates of adverse events than healthy donors (15). The analysis of efficacy of COVID-19 vaccination revealed no significant differences between PWH and healthy donors in seroconversion rates (16, 17), although most humoral studies did not consider the possible interference of some ART regimens on the neutralizing capacity of plasma IgG (18). Moreover, most studies only evaluated the humoral response, while the cellular immune response has been poorly characterized so far. The cytotoxic cellular immunity is considered an essential first line defense against SARS-CoV-2 infection (19) and the early development of cytotoxic CD8+ T cells correlates with effective viral clearance and mild disease (20, 21), being the absence of effector CD8+ T cells one major cause of COVID-19 mortality (8). Therefore, the development of cytotoxic cell populations with antiviral capacity against SARS-CoV-2 after vaccination would be essential for a full protection against COVID-19.

During HIV-1 infection, the cytotoxic response dependent on CD8+ T cells may be impaired due to several mechanisms that allow the virus to escape CD8 recognition and functionality such as MHC class I downregulation, impaired cytokine production, and alteration of TCR signaling that may induce anergy (22). These mechanisms would affect the capacity of CD8+ T cells to respond adequately to COVID-19 vaccination in PWH. However, other cells with cytotoxic activity may contribute to the general antiviral defense. NK cells are multifunctional effector cells less specialized than CD8+ T cells that may contribute to rapidly eliminate infected cells during HIV-1 acute infection (23). NK cells have a wide antiviral activity that permits the recognition and elimination of very different target cells. Although with limited capacity for immunological memory, vaccination against COVID-19 may influence the efficacy of the immune response through the stimulation of NK cells (24). Other important subset of cytotoxic cells are Tγδ cells, a lineage of unconventional T cells with broad antigen specificity and NK-like direct cytotoxic capacity that are not restricted to MHC-mediated antigen presentation (25, 26). Tγδ cell populations may change over time in response to HIV-1 infection and they are considered a surrogate marker of AIDS progression (27). Vaccination against COVID-19 appears to be able to trigger Tγδ cell populations with antiviral capacity (28).

In this prospective, longitudinal, observational study, we analyzed the changes produced in both humoral and cellular immune responses in PWH with normal CD4/CD8 ratio in response to the full vaccination schedule against COVID-19. We also characterized the main effector cells with cytotoxic activity that are mediating the cellular immune response against HIV-1 and SARS-CoV-2. This information may contribute to a better understanding about the quality of the cellular immune response in PWH and the capacity of their immune system to respond to vaccination.

## Materials and methods

### Study subjects

PWH (n=25) and healthy donors (n=16) were recruited between March and July 2021 at the Hospital Universitario Severo Ochoa (Madrid, Spain) and the Primary Healthcare Center Doctor Pedro Laín Entralgo (Madrid, Spain), respectively, for this prospective, longitudinal, observational study. Sample size was calculated using the sample size calculator Granmo (29) based on a level of confidence α=0.05 (95%) and power of the analysis β=0.2 (80%). The inclusion criteria were to be over 18 years old, CD4 > 500 cells/μl, not having a previous diagnosis of COVID-19, and to have been vaccinated through the Spanish Vaccination Program with two doses of one vaccine approved at the time for these participants: Comirnaty (BioNTech, Mainz, Germany; Pfizer, New York, NY) or Spikevax (Moderna, Cambridge, MA); or with one dose of Jcovden (Janssen, Titusville, NJ).

Blood samples were obtained before vaccination and 4–6 weeks after receiving the full vaccination schedule. Basal serology to detect IgG against SARS-CoV-2 was performed in plasma from the first blood sample to discard an asymptomatic infection before vaccination. All participants were followed up for 12 months after receiving the full vaccination schedule to record SARS-CoV-2 breakthrough infections.

### Ethical statement

All individuals gave informed written consent to participate in the study before their inclusion. Current Spanish and European Data Protection Acts guarantees the anonymity and confidentiality of all participants. Protocol for this study (CEI PI 29_2021-v3) was prepared in accordance with the Helsinki Declaration and previously reviewed and approved by the Ethics Committees of Instituto de Salud Carlos III (IRB IORG0006384) and the participating centers.

### Cells

Peripheral blood mononuclear cells (PMBCs) and plasma were isolated from whole blood collected in EDTA Vacutainer tubes (Becton Dickinson, Madrid, Spain) using Ficoll-Hypaque density gradient (Pharmacia Corporation, North Peapack, NJ) by centrifugation and then they were cryopreserved until analysis. Vero E6 cell line (African green monkey kidney; ECACC 85020206) was kindly provided by Dr. Antonio Alcami (CBM Severo Ochoa, Madrid) and it was cultured in DMEM supplemented with 10% FCS, 2 mM L-glutamine, and 100 units/ml penicillin and streptomycin. HEK-293T (embryonic human kidney; ECACC 85120602) and TZM-bl/JC53BL-13 (human cervix; NIH AIDS Research and Reference Reagent Program, no. 8129) cell lines were obtained from the existing collection of the Instituto de Salud Carlos III (Madrid, Spain) and they were cultured as described above for Vero E6 cells.

### IgG titers against SARS-CoV-2

Serology against the spike (S) protein of SARS-CoV-2 was performed in plasma of all participants using Euroimmun Anti-SARS-CoV-2 ELISA Assay (EI 2606–9601-10 G; Euroimmun, Lübeck, Germany) that can detect Alpha, Beta, Gamma, and Delta variants. Semi-quantitative results were analyzed by calculating the ratio of extinction of each plasma sample over the calibrator. Results were considered positive with IgG titer > 1.1 and negative with IgG titer < 0.8. Undermined results were located between values 1.1 and 0.8.

### SARS-CoV-2 neutralization assay

Pseudotyped single-cycle pNL4–3Δenv_SARS-CoV-2-SΔ19(G614)_Ren virus was obtained by co-transfection of HEK-293T cells with vector pNL4–3Δenv_Ren that contains HIV-1–1 genome without *env* and *renilla* luciferase gene as reporter (30), together with vector pcDNA3.1-SARS-CoV-2-SΔ19 that expresses SARS-CoV-2 *S* glycoprotein gene without the last 19 amino acids (QHU36824.1) (31). Co-transfection of pcDNA3.1-SARS-CoV-2-SΔ19 with vector pcDNA-VSV-G that expresses spike (S) glycoproteins of vesicular stomatitis virus (VSV) was used as specificity control for neutralizing assays [VSV_SARS-CoV-2-SΔ19(G614)]. The concentration of HIV-1 p24-Gag protein in the cell culture supernatants was measured 48 hours after transfection by Elecsys HIV-1 AG (Roche Diagnostic, Basel, Switzerland).

To measure the neutralization activity without ART interference, IgG were purified from plasma using Protein A HP SpinTrap (Sigma Aldrich-Merck, Darmstadt, Germany) and quantified by NanoDrop spectrophotometer (Thermo Scientific). 2-fold serial dilutions of purified IgG from IgG-positive plasma (1 to 0.008 mg/ml) were pre-incubated for 1 hour with pNL4–3Δenv_SARS-CoV-2-SΔ19(G614)_Ren pseudovirus (10ng p24-Gag per well), as previously described (32). This mixture was then added to a monolayer of Vero E6 cells and incubated for 48 hours. Viral infection in the monolayer was quantified by measuring Renilla luciferase activity (Renilla Luciferase Assay, Promega, Madison, WI) in the luminometer Centro XS3 LB 960 with MikroWin 2010 software (Berthold Technologies, Baden-Württemberg, Germany).

Titers of neutralizing antibodies were calculated as 50% inhibitory dose (NT50) which is the highest dilution of plasma that produced 50% reduction of Renilla luciferase activity in comparison with control by using a non-linear regression in GraphPad Prism Software v10.1.2. (GraphPad, Inc., San Diego, CA).

### Pseudotyped SARS-CoV-2 infection assay

Direct cellular cytotoxicity (DCC) of PBMCs was determined using Vero E6 cells infected with pNL4–3Δenv_SARS-CoV-2-SΔ19(G614)_Ren virus as target, as previously described (33). Briefly, Vero E6 cells were infected with pNL4–3Δenv_SARS-CoV-2-SΔ19(G614)_Ren (100ng p24-Gag) for 48 hours and then co-cultured for 1 hour with PBMCs from the participants (ratio 1:2). Vero E6 cell monolayer was dissociated with trypsin-EDTA (Sigma Aldrich-Merck, Darmstadt, Germany) and caspase-3 activity in these cells was measured by chemiluminescence using Caspase-Glo 3/7 Assay system (Promega, Madison, WI) and a luminometer Centro XS3 LB 960 with MikroWin 2010 software (Berthold Technologies) as a measure of PBMCs cytotoxic activity against target cells (34). PBMCs were collected from the supernatants and analyzed by flow cytometry to characterize the cytotoxic cells. The following controls were used: target cells alone as negative control, target cells infected with pseudotyped SARS-CoV-2 as basal control for viral replication, and infected target cells co-cultured with NK cell line NKL (CVCL_0466) during the same time that PBMCs from the participants as positive control for viral replication and caspase-3 activity.

### HIV-1 infection assay

TZM-bl cells infected with HIV-1 wild-type strain NL4–3 for 48 hours were used as target to evaluate the DCC of PBMCs against HIV-1. HIV-1-infected TZM-bl cells were co-cultured for 1 hour with PBMCs (ratio 1:2). TZM-bl cell monolayer was detached with Trypsin-EDTA and caspase-3 activity was measured by chemiluminescence as described above. Viral replication was determined by quantification of luciferase activity in the monolayer using the Luciferase Assay System (Promega) in the luminometer Centro XS3 LB 960 with MikroWin 2010 software (Berthold Technologies). PBMCs were collected from the supernatants and analyzed by flow cytometry to characterize the cytotoxic cells. The following controls were used: target cells alone as negative control (mock cells), target cells infected with NL4–3_wt strain as basal control for viral replication, and infected target cells co-cultured with NKL cells during the same time that PBMCs from the participants as controls for stimulation of caspase-3 activity and interference with viral replication.

### Characterization of cell populations by flow cytometry

The characterization of PBMCs that were co-cultured for 1 hour with Vero E6 and TZM-bl cell monolayers was performed by staining with the following conjugated antibodies purchased from BD Biosciences (San Jose, CA): CD3-PE, CD8-APC-H7, CD56-BV605, TCRγδ-FITC, and CD107a-PE-Cy7. CD107a was used as a degranulation marker and its expression at the cell surface peaks within 1 hour of target cell engagement (35), being afterwards actively recycled from the cell surface (36). CD3+CD8- were assumed to be CD4+ T cells, which included those cells with downregulated CD4 expression caused by HIV-1 infection (37). Isotype controls were used to determine the background signal. Data acquisition was performed with BD LSRFortessa X-20 flow cytometer (BD Biosciences) and data analysis with FlowJo software v10.0.7 (Tree Star Inc.). Gating strategy is shown in **Supplementary Figure 1**.

### Statistical analysis

All statistical analyses were performed using GraphPad Prism software v10.1.2. (GraphPad Software Inc., San Diego, CA). Data normality was assessed with Kolmogorov-Smirnov test. According to non-normal distribution of data, for comparisons within the same group in two longitudinal samples, we applied two-tailed Wilcoxon matched-pairs signed rank test. For comparisons between two independent groups, we performed unpaired, two-tailed, nonparametric Mann-Whitney U test to compare between ranks. According to data non-normality, nonparametric Spearman’s rank correlation coefficient was calculated to evaluate the correlation between the cytotoxic activity against target cells and viral replication. Simple linear regression analysis was performed using STATA 14.2 software (StataCorp LLC, College Station, TX) to estimate β regression coefficient in the comparisons between all parameters determined in PBMCs from PWH compared to healthy donors, before and after receiving the full vaccination schedule. Differences in breakthrough infections between PWH and healthy donors were evaluated by chi-square test for 2x2 contingency table using Graph Pad Prism v10.2.1. P values (p) < 0.05 were considered statistically significant in all comparisons.

## Results

### Participants’ cohorts

Sociodemographic and clinical characteristics of all participants are summarized in **Table 1** and detailed for PWH in **Supplementary Table 1**. Median age was 37 years old (Interquartile range (IQR) 33–39) in PWH and 40 years old (IQR 36–44) in healthy donors. 88% of PWH and 50% of healthy donors were male. Most participants (76% PWH and 81% healthy donors) were vaccinated with two doses of Comirnaty; 16% and 12%, respectively, received Spikevax, and 8% and 6%, respectively, received Jcovden. Median time between doses was 23 (IQR 21–33) and 21 days (IQR 21–22) for PWH and healthy donors, respectively. None of the participants in the cohort of PWH had detectable HIV-1 viremia after receiving the full vaccination schedule against SARS-CoV-2.

**Table 1:** Sociodemographic and clinical data of the participants in this study.

	PWH	Healthy donors
Participants	25	16
Demographic characteristics		
Age (years), median (IQR)	37 (33-39)	40 (36-44)
Gender (male), n (%)	22 (88)	8 (50)
Vaccination		
Comirnaty (Pfizer), n (%)	19 (76)	13 (81)
Spikevax (Moderna), n (%)	4 (16)	2 (12)
Jcovden (Janssen), n (%)	2 (8)	1 (6)
Time between doses (days), median (IQR)	23 (21-33)	21 (21-22)
Time from full vaccination to sample (days), median (IQR)	42 (27-50)	33 (29-37)
Clinical characteristics of HIV-1 infection		
Age at HIV-1 diagnosis (years), median (IQR)	29 (24-32)	NA
Time with HIV-1 infection (years), median (IQR)	6 (3-13)	NA
CD4/CD8 ratio, median (IQR)	1.0 (0.7-1.3)	2.0 (1.5-2.8)
CD4 count (cells/millilitre), median (IQR)	775 (622-998)	NA
CD8 count (cells/millilitre), median (IQR)	858 (589-1169)	NA
Viral load at the time of sampling, n, (%)		NA
Undetectable	23 (92)	
Detectable (< 2.0 log)	2 (8)	
Current ART, n (%)		NA
1 INI + 2 NRTI	13 (52)	
1 INI + 1 NRTI	6 (24)	
1 NNRTI + 2 NRTI	3 (13)	
1 NNRTI + 1 INI	1 (4.3)	
1 PI	1 (4.3)	
1 PI + 2 NRTI	1 (4.3)	
SARS-CoV-2 breakthrough infections, n (%)	8 (32)	8 (50)
Time from last vaccine dose to breakthrough infection (months), median (IQR)	5.6 (4.5-7.7)	5.3 (0.9-5.7)
Symptoms of COVID-19		
Fever, n (%)	4 (50)	0
Cough, n (%)	4 (50)	2 (25)
Dyspnea, n (%)	3 (37.5)	0

ART, Antiretroviral treatment; INI, Integrase inhibitor; IQR, Interquartile range; NA, Not Applicable; NRTI, Nucleoside reverse transcriptase inhibitor; NNRTI, Non-nucleoside reverse transcriptase inhibitor; PI, Protease inhibitor.

Median time of HIV-1 infection was 6 years (IQR 3–13) and median age at diagnosis was 29 years old (IQR 24–32). Median CD4 count was 775 (IQR 622–998) cells/µl, and median CD8 count was 858 (IQR 589–1167) for PWH. Median CD4/CD8 ratio was 1.0 (IQR 0.7–1.3) for PWH and 2.0 (IQR 1.5–2.8) for healthy donors. All PWH were on ART and the most common regimen contained three drugs of two nucleoside reverse transcriptase inhibitors (NRTIs) with one integrase inhibitor (INI) (52%), followed by two-drugs combinations of one NRTI and one INI (24%). Most PWH (92%) showed undetectable viral load at sampling.

### SARS-CoV-2 breakthrough infections

All participants were followed up for 12 months after receiving the last dose of the full vaccination schedule to record SARS-CoV-2 breakthrough infections (**Table 1**). Statistical significance in the comparison between PWH and healthy donors was determined by chi-square test (**Supplementary Figure 2**). Eight (32%) PWH and eight (50%) healthy donors had breakthrough infections with SARS-CoV-2 during this period (p=0.0097; **Supplementary Figure 2A**). Clinical data of breakthrough infections are detailed in **Table 2**. Only one (12.5%) participant from PWH cohort with CD4/CD8 of 0.68 required hospitalization due to complications of COVID-19 (p=0.0004; **Supplementary Figure 2B**). Most common symptoms during SARS-CoV-2 breakthrough infection in PWH were fever (50%), cough (50%), and dyspnea (37.5%) (p<0.001, p=0.0003, and p<0.0001, respectively), while healthy donors only reported cough (25%) as main symptom (**Supplementary Figure 2B**). Median time from the last vaccine dose to SARS-CoV-2 breakthrough infection was 5.6 months (IQR 4.5–7.7) and 5.3 months (IQR 0.9–5.7), respectively. Most individuals with breakthrough infections from both cohorts were vaccinated with Comirnaty (87.5% for both PWH and healthy donors), which was the vaccine most administered to the participants.

**Table 2:** Clinical data of SARS-CoV-2 breakthrough infections and COVID-19 vaccination of all participants in this study.

Participant’s ID	Demographical data		HIV infection (+/-)	SARS-CoV-2 Vaccines	SARS-CoV-2 breakthrough infection					
	Age (years)	Gender (M/F)			Time from last vaccine dose to SARS-CoV-2 diagnosis in case of infection (days)	Hospitalization (Y/N)	Fever (Y/N)	Cough (Y/N)	Dyspnea (Y/N)	Pneumonia (Y/N)
1	45	F	+	Comirnaty	-	-	-	-	-	-
2	42	M	+	Comirnaty	244	N	Y	Y	Y	N
3	38	M	+	Comirnaty	159	N	Y	Y	N	N
4	35	M	+	Comirnaty	-	-	-	-	-	-
5	40	M	+	Jcovden	-	-	-	-	-	-
6	40	M	+	Comirnaty	-	-	-	-	-	-
7	39	M	+	Spikevax	-	-	-	-	-	-
8	35	M	+	Comirnaty	172	N	N	N	N	N
9	39	M	+	Jcovden	-	-	-	-	-	-
10	28	M	+	Comirnaty	-	-	-	-	-	-
11	49	F	+	Comirnaty	125	N	N	N	Y	N
12	35	M	+	Comirnaty	-	-	-	-	-	-
13	21	M	+	Spikevax	-	-	-	-	-	-
14	32	M	+	Comirnaty	-	-	-	-	-	-
15	31	M	+	Comirnaty	331	N	N	Y	N	N
16	39	F	+	Comirnaty	162	N	Y	Y	Y	N
17	38	M	+	Comirnaty	-	-	-	-	-	-
18	38	M	+	Comirnaty	95	N	N	N	N	N
19	34	M	+	Comirnaty	-	-	-	-	-	-
20	35	M	+	Comirnaty	-	-	-	-	-	-
21	44	M	+	Comirnaty	-	-	-	-	-	-
22	33	M	+	Spikevax	-	-	-	-	-	-
23	37	M	+	Comirnaty	-	-	-	-	-	-
24	33	M	+	Comirnaty	-	-	-	-	-	-
25	27	M	+	Spikevax	192	Y	Y	N	N	N
26	47	M	–	Comirnaty	-	-	-	-	-	-
27	40	M	–	Comirnaty	-	-	-	-	-	-
28	35	F	–	Comirnaty	26	N	N	N	N	N
29	40	F	–	Comirnaty	5	N	N	N	N	N
30	55	F	–	Spikevax	–					
31	55	F	–	Spikevax	-	-	-	-	-	-
32	44	F	–	Jcovden	33	N	N	N	N	N
33	30	M	–	Comirnaty	158	N	N	Y	N	N
34	30	F	–	Comirnaty	168	N	N	N	N	N
35	36	M	–	Comirnaty	358	N	N	N	N	N
36	45	M	–	Comirnaty	-	-	-	-	-	-
37	39	M	–	Comirnaty	157	N	N	Y	N	N
38	37	F	–	Comirnaty	-	-	-	-	-	-
39	42	M	–	Comirnaty	172	N	N	N	N	N
40	25	F	–	Comirnaty	-	-	-	-	-	-
41	44	M	–	Comirnaty	-	-	-	-	-	-

F, Female; M, Male; N/A, not applicable; N, No; Y, Yes.

### Blood samples

The first blood sample was collected before receiving the first vaccine dose. A basal serology against SARS-CoV-2 was performed in this sample to identify and exclude those individuals with previous asymptomatic COVID-19. Median time since the participants received the full vaccination schedule and the collection of the second blood sample was 42 (IQR 27–50) and 33 days (IQR 29–37), respectively, in PWH and healthy donors (**Table 1**).

### High IgG levels against SARS-CoV-2 and neutralizing capacity of plasma from PWH in response to vaccination

**Figure 1:**
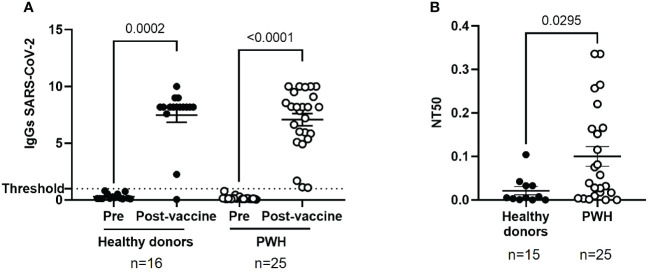
Humoral response against SARS-CoV-2 in PWH before and one month after receiving the complete vaccination schedule against COVID-19. **(A)** Levels of total IgG against SARS-CoV-2 in plasma of PWH and healthy donors, before and after receiving the complete vaccination schedule. Positive threshold (dotted line) indicates positive IgG titers above 1.1. **(B)** Neutralizing capacity of purified, specific IgG in seropositive individuals one month after receiving the complete vaccination schedule, represented as 50% inhibitory dose (NT50). Each dot corresponds to one sample and lines represent the mean ± standard error of the mean (SEM). Each symbol represents a different cohort: healthy donors (closed circles) and PWH (open circles). Wilcoxon signed-rank test was applied to calculate the statistical significance within groups. Mann-Whitney U test was applied to calculate the statistical significance between unpaired groups.

The analysis of IgG levels against SARS-CoV-2 revealed an excellent humoral response of PWH after receiving the full vaccination schedule, which was comparable to healthy donors (p<0.0001 and p=0.0002, respectively) (**Figure 1A**).

The neutralizing capacity of plasma isolated from PWH was evaluated by using pseudotyped NL4–3Δenv_SARS-CoV-2-SΔ19(G614)_Ren virus to infect Vero E6 as target cells, as well as pseudotyped VSV_SARS-CoV-2-SΔ19(G614) virus to determine the presence of unspecific reaction, as described previously (33). We observed a high non-specific neutralization activity of VSV_SARS-CoV-2-SΔ19(G614)_Ren virus in plasma from PWH (**Supplementary Figure 3**) that has been previously related to interference with ART based on integrase inhibitors (18). To avoid false positive neutralization results, the neutralizing capacity against SARS-CoV-2 was analyzed after purification of IgG from plasma of PWH and healthy donors. The neutralizing capacity of IgG purified from plasma of PWH was 4.7-fold higher than healthy donors (p=0.0295) (**Figure 1B**).

### Enhanced cytotoxic activity against SARS-CoV-2-infected cells in PBMCs from PWH

**Figure 2:**
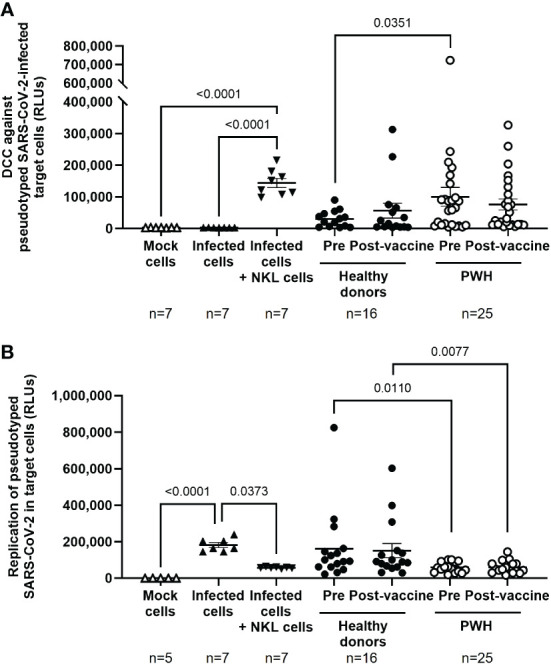
Cytotoxic activity of PBMCs from PWH against SARS-CoV-2 before and one month after receiving the complete vaccination schedule against COVID-19. **(A)** Direct cellular cytotoxicity (DCC) of PBMCs isolated from PWH and healthy donors was determined as induced caspase-3 activity in pseudotyped SARS-CoV-2-infected Vero E6 cells measured by chemiluminescence (RLUs, Relative Light Units). **(B)** Replication of pseudotyped SARS-CoV-2 in infected Vero E6 cells in the presence of PBMCs from PWH and healthy donors, measured by luminescence as the production of Renilla (RLUs). Each dot corresponds to one sample and lines represent the mean ± SEM. Each symbol represents a different cohort: healthy donors (closed circles) and PWH (open circles). Target cells alone (mock cells) were used as negative control (open triangles), target cells infected with pseudotyped SARS-CoV-2 were used as basal control for viral replication (closed triangles), and infected target cells co-cultured with NKL cells were used as positive control for viral replication and caspase-3 activity (inverted closed triangles). Wilcoxon signed-rank test was applied to calculate the statistical significance within groups. Mann-Whitney U test was applied to calculate the statistical significance between unpaired groups. Statistical significances between controls and sample groups are not shown.

DCC of PBMCs from PWH against Vero E6 cells infected with pseudotyped NL4–3Δenv_SARS-CoV-2-SΔ19(G614)_Ren virus was 3.3-fold (p=0.0351) higher in the basal sample than in PBMCs from healthy donors (**Figure 2A**). Accordingly, viral replication in Vero E6 cells was reduced 2.7-fold (p=0.0110) in the presence of PBMCs from PWH previously unexposed to SARS-CoV-2 (**Figure 2B**). This difference was still significant after receiving the full vaccination schedule (-2.6-fold; p=0.0077). Calculation of the Spearman’s rank correlation coefficient showed a significant negative correlation between DCC and viral replication in SARS-CoV-2-infected target cells in the presence of PBMCs from PWH (r=-0.5059; p=0.0479) and healthy donors (r=-0.5294; p=0.0372) after receiving the full vaccination schedule, and before vaccination in PBMCs from PWH (r=-0.5088; p=0.0464), but not from healthy donors (**Supplementary Figure 4**).

### Increased levels of CD8+ T cells and Tγδ cell populations with degranulation capacity in PBMCs from PWH

**Figure 3:**
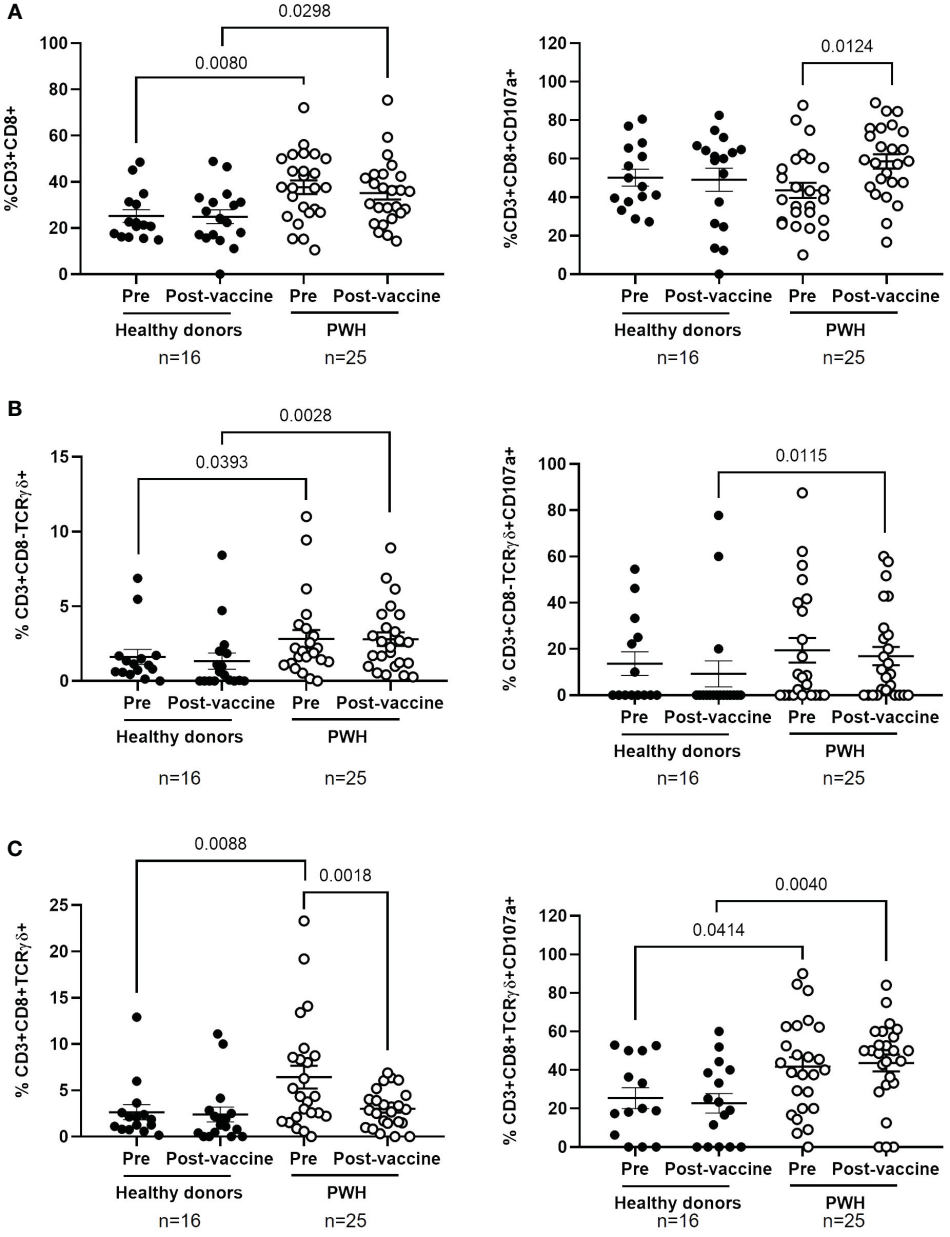
Analysis of cytotoxic cell populations in PBMCs from PWH before and one month after receiving the complete vaccination schedule against COVID-19. Levels of CD8+ T cells **(A)** and Tγδ cell populations with phenotypes CD3+CD8-TCRγδ+ **(B)** and CD3+CD8+TCRγδ+ **(C)** (left graphs) in PBMCs from PWH and healthy donors, as well as the expression of CD107a on their surface (right graphs) in response to co-culture with pseudotyped SARS-CoV-2-infected Vero E6 cells. Each dot corresponds to one sample and lines represent the mean ± SEM. Each symbol represents a different cohort: healthy donors (closed circles) and PWH (open circles). Wilcoxon signed-rank test was applied to calculate the statistical significance within groups. Mann-Whitney U test was applied to calculate the statistical significance between groups.

No significant differences between PWH and healthy donors were observed in the levels of CD4+ T cells before and after the administration of COVID-19 vaccines (**Supplementary Figure 5**), but the levels of CD8+ T cells in PBMCs from PWH were higher than healthy donors before and after receiving the full vaccination schedule (1.5-fold, p=0.0080 and 1.4-fold, p=0.0298, respectively) (**Figure 3A**, left graph). The degranulation capacity of these cells, determined by the expression levels of CD107a of the cell surface, was similar between both cohorts but it was significantly higher in PWH after receiving the full vaccination schedule (1.3-fold; p=0.0124) (**Figure 3A**, right graph).

The levels of Tγδ cell populations with phenotypes CD3+CD8-TCRγδ+ and CD3+CD8+TCRγδ+ were higher in the basal sample of PBMCs from PWH than in healthy donors (1.8-fold, p=0.0393 and 2.6-fold, p=0.0088, respectively) (**Figures 3B, C**, left graphs). The levels of CD8- Tγδ cells were still higher in PWH than in healthy donors after receiving the full vaccination schedule (3.9-fold; p=0.0028). Within cohorts, the levels of CD8- Tγδ cells did not change after receiving the full vaccination schedule in either group, while the levels of CD8+ Tγδ cells were reduced 2.0-fold (p=0.0018) in PWH after receiving vaccination. The degranulation capacity of Tγδ cell populations was significantly higher in PBMCs from PWH than in healthy donors, and these differences achieved statistical significance in the comparison after receiving the full vaccination schedule (1.8-fold, p=0.0115 and 2.0-fold, p=0.0040, respectively) (**Figures 3B, C**, right graphs). In PWH, CD8+ Tγδ cells also showed higher degranulation capacity than healthy donors in response to infected cells before vaccination (1.6-fold; p=0.0414) (**Figure 3C**, right graph).

We did not find significant differences in the levels of NK and NKT cells in PBMCs from both cohorts, before and after receiving the full vaccination schedule, or in the expression levels of CD107a in these cell populations (**Supplementary Figure 6**). The effect of COVID-19 vaccination on the levels of CD8+ T cells and Tγδ cells expressing CD56 on the cell surface was also analyzed. After vaccination, the levels of CD3+CD8+CD56+ cells increased 1.8-fold (p=0.0462) in healthy donors, in comparison with PWH (**Supplementary Figure 7A**, left graph). No changes in the expression of CD56 in Tγδ CD8+ or CD8- cells were observed between PWH and healthy donors (**Supplementary Figures 7B, C**, left graph). In addition, the expression of CD107a increased 2.0-fold (p=0.0142) in Tγδ CD8+CD56+ from PWH, in comparison with healthy donors, after vaccination (**Supplementary Figure 7C**, right graph).

### Simple linear regression analysis revealed the presence of cellular immunity against SARS-CoV-2 in PBMCs from PWH before vaccination

Simple linear regression analysis was performed to estimate β regression coefficient in the comparisons between all parameters involved in cellular immunity determined in PBMCs from PWH compared to healthy donors before and after receiving the full vaccination schedule. Before vaccination, the average difference between DCC of PBMCs from PWH in comparison with healthy donors was positive (β=69957.19; p=0.050), while the average difference for SARS-CoV-2 replication from infected target cells was negative (β=-101924.30; p=0.009) (**Supplementary Table 2A**). In addition, the average difference for the levels of CD8+ T cells (β=12.4840; p=0.003), Tγδ CD8+ cells (β=5.7361; p=0.043), and their degranulation capacity (Tγδ CD8+CD107a cells) (β=16.3553; p=0.021) was also positive in the comparison between PWH and healthy donors. These results supported the notion that PWH had cellular immunity with capacity to eliminate SARS-CoV-2-infected cells before being exposed to the virus or vaccine. After vaccination, the average difference between the ability of PBMCs from PWH to eliminate SARS-CoV-2-infected target cells in comparison with healthy donors was still negative (β=-90834.43; p=0.004), while the average difference for the levels of CD8+ T cells (β=10.2614; p=0.016), Tγδ CD8- cells (β=1.4706; p=0.034), and Tγδ CD8+CD107a cells (β=20.8745; p=0.002) was still positive (**Supplementary Table 2B**).

### Full vaccination against SARS-CoV-2 did not affect the cellular immune response against HIV-1 in PWH

**Figure 4:**
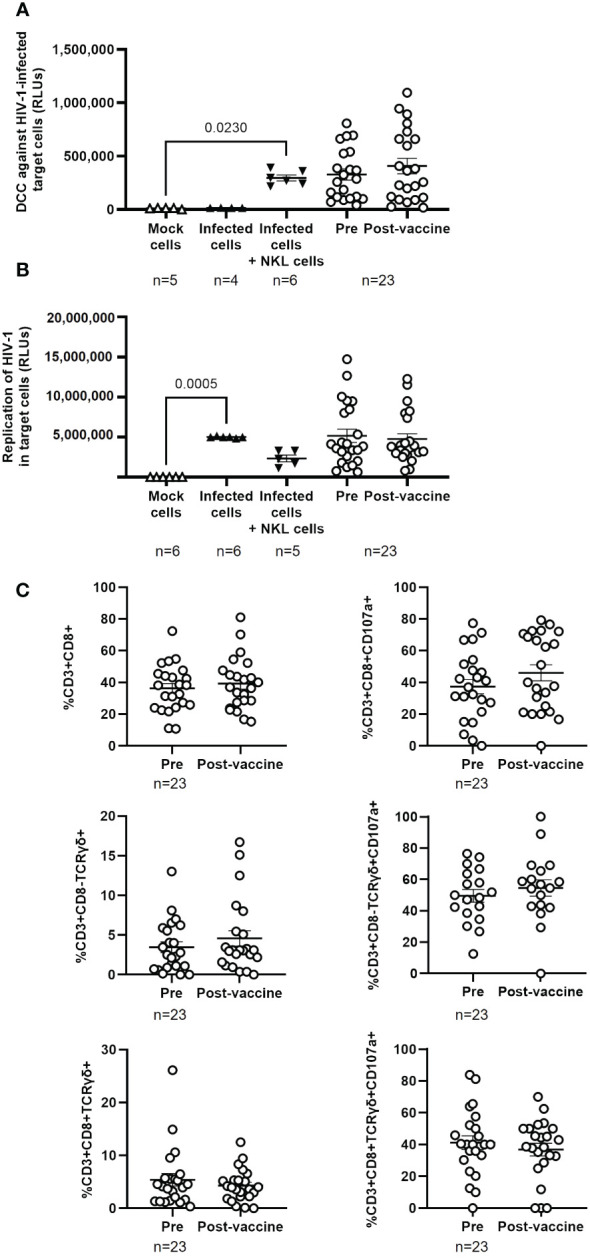
Cytotoxic activity of PBMCs from PWH against HIV-1 before and one month after receiving the complete vaccination schedule against COVID-19. **(A)** Direct cellular cytotoxicity (DCC) of PBMCs isolated from PWH was determined as induced caspase-3 activity in HIV-1-infected TZM-bl cells measured by chemiluminescence (RLUs). **(B)** Replication of HIV-1 in infected TZM-bl cells in the presence of PBMCs from PWH, measured by luminescence as the production of Renilla (RLUs). **(C)** Levels of cytotoxic cell populations (left graphs) and the expression of CD107a on the cell surface (right graphs) in response to co-culture with HIV-1-infected TZM-bl cells. Each dot corresponds to one sample and lines represent the mean ± SEM. Target cells alone (mock cells) were used as negative control (open triangles), target cells infected with NL4–3_wt strain were used as basal control for viral replication (closed triangles), and infected target cells co-cultured with NKL cells were used as positive control for viral replication and caspase-3 activity (inverted closed triangles). Wilcoxon signed-rank test was applied to calculate the statistical significance. Statistical significances between controls and sample groups are not shown.

DCC against TZM-bl cells infected with NL4–3 wild-type strain was evaluated in PBMCs from PWH to determine the effect of the full vaccination schedule against SARS-CoV-2 in the immune response against HIV-1. DCC response was higher against target cells infected with HIV-1 than with pseudotyped SARS-CoV-2 (see **Figure 2A**), but no significant changes in DCC against HIV-1-infected cells were observed before and after vaccination against SARS-CoV-2 (**Figure 4A**) or on the viral replication in the target infected cells (**Figure 4B**). No changes were observed either in the levels of CD8+ T cells and Tγδ CD8+ or CD8- cells (**Figure 4C**, left graphs) or in the expression of the degranulation marker CD107a after vaccination in the response against HIV-1 infected target cells (**Figure 4C**, right graphs). Calculation of the Spearman’s rank correlation coefficient showed a significant negative correlation between DCC and viral replication in HIV-1-infected target cells in the presence of PBMCs from PWH before (r=-0.4289; p=0.0412) and after receiving the full vaccination schedule (r=-0.4444; p=0.0383) (**Supplementary Figure 8**).

## Discussion

PWH may be more susceptible to develop severe forms of infectious diseases due to the damage caused by HIV-1 in the host immune system (38, 39). However, there is still no consensus whether SARS-CoV-2 infection has a worse outcome in PWH in comparison with healthy population (4, 40–42), most likely because there are several types of HIV-1 disease progression depending on the condition of the immune system (43). It has been suggested that CD4/CD8 ratio may be useful to monitor HIV-1 infection and to improve clinical decision making. Although there is not clear increase in the rate of COVID-19 among PWH in comparison with the general population, low levels of CD4+ T cells have been related to severe forms of the disease (44), and low or inverted CD4/CD8 ratio reflects a decreased capacity to respond to SARS-CoV-2 (45). Accordingly, PWH with normal CD4/CD8 ratio should be able to respond efficiently to vaccination against COVID-19.

In our cohort of PWH, who were mostly male with undetectable viremia, high levels of total IgG against the S protein of SARS-CoV-2 were detected in plasma in response to vaccination, similar to healthy donors, which was in accordance with previous reports (46–50). Only two PWH with CD4 count below 200 cells/μl produced low levels of IgG in response to vaccination, as was described previously (47). Most (59.6%) participants in our study were vaccinated with Comirnaty, which was the most administered vaccine in Spain at the time of sample collection, followed by Spikevax (21.4%) (51). We did not find significant differences between IgG titers elicited by Comirnaty and Spikevax. However, IgG titers of participants vaccinated with Jcovden were barely above the limit considered positive, in accordance with previous studies (52).

It has been described that plasma from PWH with CD4 count above 200 cells/μl usually presents a similar neutralizing capacity to healthy donors (17, 32, 53–55). However, we observed a significantly higher neutralizing capacity of IgG in plasma of PWH than in healthy donors. This discrepancy could be related to the unspecific response that was observed when total plasma was analyzed to determine the neutralizing capacity instead of purified IgG. Due to ART regimens including integrase inhibitors may cause an unspecific neutralizing response (18), our results are likely more accurate than others previously published in which pseudotyped VSV SARS-CoV-2 virus was not used as control of specificity or total plasma was analyzed.

After determining that the humoral response was effective in PWH with normal CD4/CD8 ratio in response to SARS-CoV-2 vaccines, the main objective of our study was to evaluate the cellular immune response and the cell populations that were developed after receiving the complete vaccination schedule against COVID-19. Interestingly, before vaccination, PBMCs from PWH showed a potent cytotoxic activity against SARS-CoV-2-infected cells that was higher than healthy donors previously unexposed to the virus. This cytotoxic response was efficient to control SARS-CoV-2 replication in infected target cells and it was not due to a previous asymptomatic infection with SARS-CoV-2 as all participants in the study were seronegative upon recruitment. PWH present a chronic immune activation state due to HIV-1 persistence that rarely returns to normal levels even in the presence of ART (56) and that could contribute to the effective cytotoxic response against SARS-CoV-2 observed before vaccination. When PBMCs from PWH that were co-cultured with SARS-CoV-2-infected target cells were analyzed, we observed higher levels of functional CD8+ T cells than in healthy donors before vaccination against COVID-19 that were maintained after vaccination. Within six months of HIV-1 seroconversion, CD8 count usually increases about 40% while CD4 count is reduced about 30%, resulting in an inverted CD4/CD8 ratio below 1.0 (57). The expansion of clonal CD8+ T cells specific of HIV-1 that may invert CD4/CD8 ratio is common in PWH, and it has also been described in response to cytomegalovirus infection (58), tuberculosis and toxoplasmosis (59), as well as during the immune response against SARS-CoV-2. In the absence of a previous exposure to SARS-CoV-2, the pre-existence of functional CD8+ T cell populations with antiviral activity against SARS-CoV-2 cannot be ruled out as they have been described in health workers who presented abortive, seronegative infection at the beginning of COVID-19 pandemic (60). These T cell populations were specific of the viral polymerase, and they expanded and accumulated before exposure to SARS-CoV-2, interfering with the establishment of SARS-CoV-2 infection. Other reports also described functional pre-existing SARS-CoV-2 cross-reactive memory T cells in the general population that were present in the organism previous to the viral exposure (61–67), as well as against other viruses such as influenza (68) and hepatitis B virus (69), or even against viral vectors used during gene therapy and immunotherapy (70–74). However, there are no reports that pre-existing T cell responses against SARS-CoV-2 may be present in PWH with normal CD4/CD8 ratio. In our cohort of PWH, the level of CD8+ T cells was higher in PWH than healthy donors before and after vaccination, which may be related to HIV-1 persistence, but the degranulation capacity of these cells in response to SARS-CoV-2-infected cell was similar to healthy donors before vaccination and increased after vaccination. These results proved that the potency of CD8-related cytotoxic response may be enhanced in PWH in response to COVID-19 vaccination. However, other cells were responsible for the enhanced cytotoxicity in PWH previously unexposed to SARS-CoV-2.

Many other immune cell types have been associated with HIV-1-induced immune activation (75). In addition to CD8+ T cells, we observed high levels of Tγδ cells with degranulation capacity in PBMCs from PWH before vaccination, both CD3+CD8-TCRγδ+ and CD3+CD8+TCRγδ+ phenotypes. Tγδ cells display a potent cytotoxic activity, they have a protective role in cancer, and they are essential in the first line response to viral infections (76, 77). Tγδ cells may be activated by stress signals from infected or tumor cells (78). Vaccination may boost not only adaptive immunity but also innate immunity, including Tγδ cells (79), but in our cohort of PWH the levels of these cells were high before COVID-19 vaccination. Tγδ cells have been found increased in PWH with normal CD4/CD8 ratio and they may control HIV-1 replication through several mechanisms, including DCC of infected cells (80). In fact, ART promotes the recovery of Tγδ polyfunctionality, as well as the TCR repertoire diversity and cytotoxic activity (81). The role of Tγδ cells has also been described during SARS-CoV-2 infection, demonstrating that the activation of these cells may lead to inhibition of SARS-CoV-2 replication (82, 83). Consequently, this is the first report that pre-existing Tγδ cells may also contribute to protect PWH with normal CD4/CD8 ratio from other viral pathogens such as SARS-CoV-2, even before vaccination. Both CD8+ and CD8- Tγδ cells from PWH maintained higher degranulation capacity before and after vaccination. Although pre-existing NK cell responses against SARS-CoV-2 in pre-pandemic serum from PWH have also been described (84), in our cohort NK and NKT cells appeared not to be contributing to the increased cytotoxic activity in PWH before vaccination.

One potential limitation of this study is that the functionality of CD4+ T cells was not determined directly, and these cells may be altered in PWH due to the viral infection (85). However, CD4/CD8 ratio is considered the best biomarker for overall immune functionality (59) and the median of this ratio in our cohort of PWH was 1.0 and only three participants showed CD4 counts below 500 cells/μl. Moreover, impaired CD4 functionality may affect both humoral and cellular responses and causes immunosuppression (86), but all participants in our cohort of PWH developed specific IgG against SARS-CoV-2 after vaccination and their PBMCs showed functional cytotoxic activity with capacity to reduce viral replication from SARS-CoV-2-infected cells, even those from individuals with low CD4 counts. These results are in accordance with Bessen et al. (87) who describe that low CD4 counts do not necessarily interfere with CD4 functionality and optimum cellular immune responses. In addition, none of the participants in our cohort of PWH presented opportunistic infections, which proved that their immune system was competent, and consequently, that their CD4+ T cells were not dysfunctional. On the other hand, we mostly recruited male participants for this study due to in Spain the incidence ratio of men to women is approximately 5 and only one of every 10 new cases is a woman (88). Finally, the immunity against SARS-CoV-2 developed by Comirnaty, Spikevax, and Jcovden vaccines may be different to other vaccines that are currently available.

In conclusion, we determined that PWH with normal CD4/CD8 ratio showed an excellent humoral response to COVID-19 vaccination based on high levels of IgG against SARS-CoV-2 S protein with enhanced neutralizing capacity in comparison with healthy donors. The full vaccination schedule induced a higher degranulation capacity in response to SARS-CoV-2 infected cells in CD8+ T cells from PWH but not from healthy donors. In addition, we observed a potent cytotoxic activity against SARS-CoV-2 before vaccination in PWH that was supported by high levels of pre-existing highly cytotoxic Tγδ CD8+ or CD8- cells with degranulation capacity. This precedent cellular immunity could be related to the low incidence of mild SARS-CoV-2 breakthrough infections in our cohort of PWH that was significantly lower than healthy donors, although PWH showed more symptoms during COVID-19, likely due to the detrimental effects of HIV-1 persistence on the immune response. Therefore, the monitorization of PWH is necessary to evaluate possible changes over time in the protective immunity against SARS-CoV-2 caused by the sustained immune activation. More studies will be necessary to determine the presence of these pre-existing cytotoxic cells in PWH with unfavorable CD4/CD8 ratio or its persistence over time.

## Data availability statement

The original contributions presented in the study are included in the article/**Supplementary Material**. Further inquiries can be directed to the corresponding author.

## Ethics statement

The studies involving humans were approved by Ethics Committees of Instituto de Salud Carlos III (IRB IORG0006384). The studies were conducted in accordance with the local legislation and institutional requirements. The participants provided their written informed consent to participate in this study.

## Author contributions

GC-F: Formal Analysis, Investigation, Methodology, Writing – original draft, Writing – review & editing. JC: Formal Analysis, Investigation, Resources, Writing – review & editing, Methodology. LN: Formal Analysis, Methodology, Writing – review & editing. FR-M: Investigation, Methodology, Writing – review & editing. MM: Methodology, Resources, Writing – review & editing. CS-M: Investigation, Methodology, Writing – review & editing. DF: Data curation, Formal analysis, Methodology, Validation, Writing – original draft, Writing – review & editing. EM: Investigation, Methodology, Writing – review & editing. MAM-A: Methodology, Resources, Writing – review & editing. MP-O: Investigation, Methodology, Writing – review & editing. MiC: Formal Analysis, Methodology, Writing – review & editing. MT: Data curation, Formal Analysis, Investigation, Methodology, Writing – review & editing. RR-S: Data curation, Funding acquisition, Investigation, Resources, Writing – review & editing. MaC: Conceptualization, Data curation, Formal Analysis, Funding acquisition, Supervision, Validation, Writing – original draft, Writing – review & editing, Methodology.
