# Supplementary material for: Pre-existing cell populations with cytotoxic activity against SARS-CoV-2 in people with HIV and normal CD4/CD8 ratio previously unexposed to the virus
Source: Front Immunol. 2024 May 15;15:1362621. doi: 10.3389/fimmu.2024.1362621 (PMC11133563; doi:10.3389/fimmu.2024.1362621)
Supplement: Supplementary file 1 [file Table_1.docx]

**Supplemental Table 1.** Sociodemographic and clinical data of PWH recruited for this study.

| **Participant’s**  **ID** | **Demographical data** | | **HIV-1 infection data** | | | | | | | | | | |
| --- | --- | --- | --- | --- | --- | --- | --- | --- | --- | --- | --- | --- | --- |
|  | **Age (years)** | **Gender**  **(M/F)** | **Route of infection (S/P)** | **Age at HIV-1 diagnosis (years)** | **Time with HIV-1 infection (years)** | **Viral load at diagnosis**  **(log)** | **Viral load**  **(log)** | **CD4 count (cells/mL)** | **Nadir CD4 (cells/mL)** | **CD8 count (cells/mL)** | **Ratio CD4/CD8** | **Current ART** | **Opportunistic disease** |
| 1 | 45 | F | S | 24 | 22 | 2.2 | U | 891 | 140 | 1071 | 0.83 | 1 NNRTI + 2 NRTI | No |
| 2 | 42 | M | S | 38 | 4 | 4.6 | U | 897 | 613 | 449 | 2.00 | 1 INI + 2 NRTI | No |
| 3 | 38 | M | S | 29 | 9 | 4.9 | U | 864 | 479 | 861 | 1.00 | 1 INI + 1 NRTI | No |
| 4 | 35 | M | S | 28 | 8 | 3.5 | U | 775 | 598 | 616 | 1.26 | 1 INI + 2 NRTI | No |
| 5 | 40 | M | S | 25 | 15 | 4.5 | U | 1174 | 420 | 1182 | 0.99 | 1 NNRTI + 1 INI | No |
| 6 | 40 | M | S | 36 | 4 | 4.7 | U | 653 | 254 | 900 | 0.73 | 1 INI + 2 NRTI | No |
| 7 | 39 | M | S | 27 | 12 | 4.8 | U | 665 | 320 | 1322 | 0.50 | 1 INI + 2 NRTI | No |
| 8 | 35 | M | S | 33 | 3 | 5.0 | U | 1072 | 441 | 1167 | 0.92 | 1 INI + 2 NRTI | No |
| 9 | 39 | M | S | 32 | 8 | 4.8 | U | 1439 | 524 | 967 | 1.49 | 1 INI + 2 NRTI | No |
| 10 | 28 | M | S | 19 | 10 | 3.0 | U | 998 | 868 | 1032 | 0.97 | 1 NNRTI + 2 NRTI | No |
| 11 | 49 | F | S | 29 | 20 | 6.1 | U | 986 | 44 | 400 | 2.47 | 1 PI | No |
| 12 | 35 | M | S | 30 | 5 | 2.2 | U | 754 | 587 | 1171 | 0.64 | 1 INI + 2 NRTI | No |
| 13 | 21 | M | S | 19 | 2 | 4.5 | U | 643 | 509 | 513 | 1.25 | 1 PI + 2 NRTI | No |
| 14 | 32 | M | S | 1 | 32 | 3.9 | 1.8 | 124 | 55 | 1485 | 0.08 | 1 INI + 2 NRTI | No |
| 15 | 31 | M | S | 29 | 2 | 3.5 | U | 544 | 515 | 440 | 1.24 | 1 INI + 1 NRTI | No |
| 16 | 39 | F | S | 23 | 16 | 2.9 | U | 618 | 199 | 858 | 0.72 | 1 INI + 1 NRTI | No |
| 17 | 38 | M | S | 35 | 3 | 2.5 | U | 947 | 505 | 478 | 1.98 | 1 INI + 2 NRTI | No |
| 18 | 38 | M | S | 32 | 6 | 4.7 | U | 1676 | 806 | 528 | 3.17 | 1 INI + 1 NRTI | No |
| 19 | 34 | M | S | 29 | 5 | 4.6 | U | 1348 | 790 | 2036 | 0.66 | 1 INI + 1 NRTI | No |
| 20 | 35 | M | S | 23 | 13 | 5.5 | U | 640 | 340 | 1816 | 0.35 | 1 INI + 2 NRTI | No |
| 21 | 44 | M | S | 30 | 15 | 4.5 | U | 1112 | 279 | 825 | 1.35 | 1 NNRTI + 2 NRTI | No |
| 22 | 33 | M | S | 23 | 1 | 5.4 | 1.7 | 168 | 168 | 682 | 0.25 | 1 INI + 2 NRTI | No |
| 23 | 37 | M | S | 32 | 6 | 4.7 | U | 431 | 233 | 589 | 0.73 | 1 INI + 2 NRTI | No |
| 24 | 33 | M | S | 31 | 2 | 3.7 | U | 622 | 463 | 613 | 1.00 | 1 INI + 2 NRTI | No |
| 25 | 27 | M | S | 26 | 1 | 3.9 | U | 511 | 282 | 756 | 0.68 | 1 INI + 1 NRTI | No |

ART. Antiretroviral treatment; F, Female; INI. Integrase inhibitor; IQR. Interquartile range; M, Male; NA. Not Applicable; NRTI. Nucleoside reverse transcriptase inhibitor; NNRTI. Non-nucleoside reverse transcriptase inhibitor; P, Parenteral; PI. Protease inhibitor; S, Sexual; U: undetectable.
