# Supplementary material for: Pre-existing cell populations with cytotoxic activity against SARS-CoV-2 in people with HIV and normal CD4/CD8 ratio previously unexposed to the virus
Source: Front Immunol. 2024 May 15;15:1362621. doi: 10.3389/fimmu.2024.1362621 (PMC11133563; doi:10.3389/fimmu.2024.1362621)
Supplement: Supplementary file 2 [file Table_2.docx]

**Supplemental Table 2**. Linear regression analysis of immune parameters in PWH versus healthy donors before (A) and after (B) receiving the full vaccination schedule against COVID-19.

**A**

| Parameter | β | p-value | 95% CI |
| --- | --- | --- | --- |
| IgG | -0.1341 | **0.022** | -0.2477 to -0.0204 |
| DCC | 69957.19 | **0.050** | -1103.45 to 141017.80 |
| SARS-CoV-2 replication | -101924.30 | **0.009** | -176459.10 to -27389.64 |
| CD8+ T cells | 12.4840 | **0.003** | 4.4567 to 20.5113 |
| CD8+CD107a+ | -6.5466 | 0.245 | -17.7618 to 4.6684 |
| Tγδ CD8- | 2.4704 | 0.205 | -1.4009 to 6.3417 |
| Tγδ CD8-CD107a+ | 1.8076 | 0.797 | -12.3220 to 15.9372 |
| Tγδ CD8+ | 5.7361 | **0.043** | 0.1900 to 11.2821 |
| Tγδ CD8+CD107a+ | 16.3553 | **0.021** | 2.6070 to 30.1036 |

**B**

| Parameter | β | p-value | 95% CI |
| --- | --- | --- | --- |
| IgG | -0.4059 | 0.615 | -2.0240 to 1.2121 |
| Neutralizing IgG | -0.5590 | 0.484 | -0.2158 to 0.1039 |
| DCC | 22957.91 | 0.391 | -30466.61 to 76382.42 |
| SARS-CoV-2 replication | -90834.43 | **0.004** | -151017.30 to -30651.52 |
| CD8+ T cells | 10.2614 | **0.016** | 2.0053 to 18.5174 |
| CD8+CD107a+ | 9.5352 | 0.151 | -3.6157 to 22.6863 |
| Tγδ CD8- | 1.4706 | **0.034** | 0.1134 to 2.8279 |
| Tγδ CD8-CD107a+ | 7.6132 | 0.247 | -5.4811 to 20.7076 |
| Tγδ CD8+ | 0.6270 | 0.435 | -0.9780 to 2.2322 |
| Tγδ CD8+CD107a+ | 20.8745 | **0.002** | 7.8918 to 33.8571 |
