# Supplementary material for: Pre-existing cell populations with cytotoxic activity against SARS-CoV-2 in people with HIV and normal CD4/CD8 ratio previously unexposed to the virus
Source: Front Immunol. 2024 May 15;15:1362621. doi: 10.3389/fimmu.2024.1362621 (PMC11133563; doi:10.3389/fimmu.2024.1362621)
Supplement: Supplementary file 3 [file Presentation_1.pptx]

## Slide 1
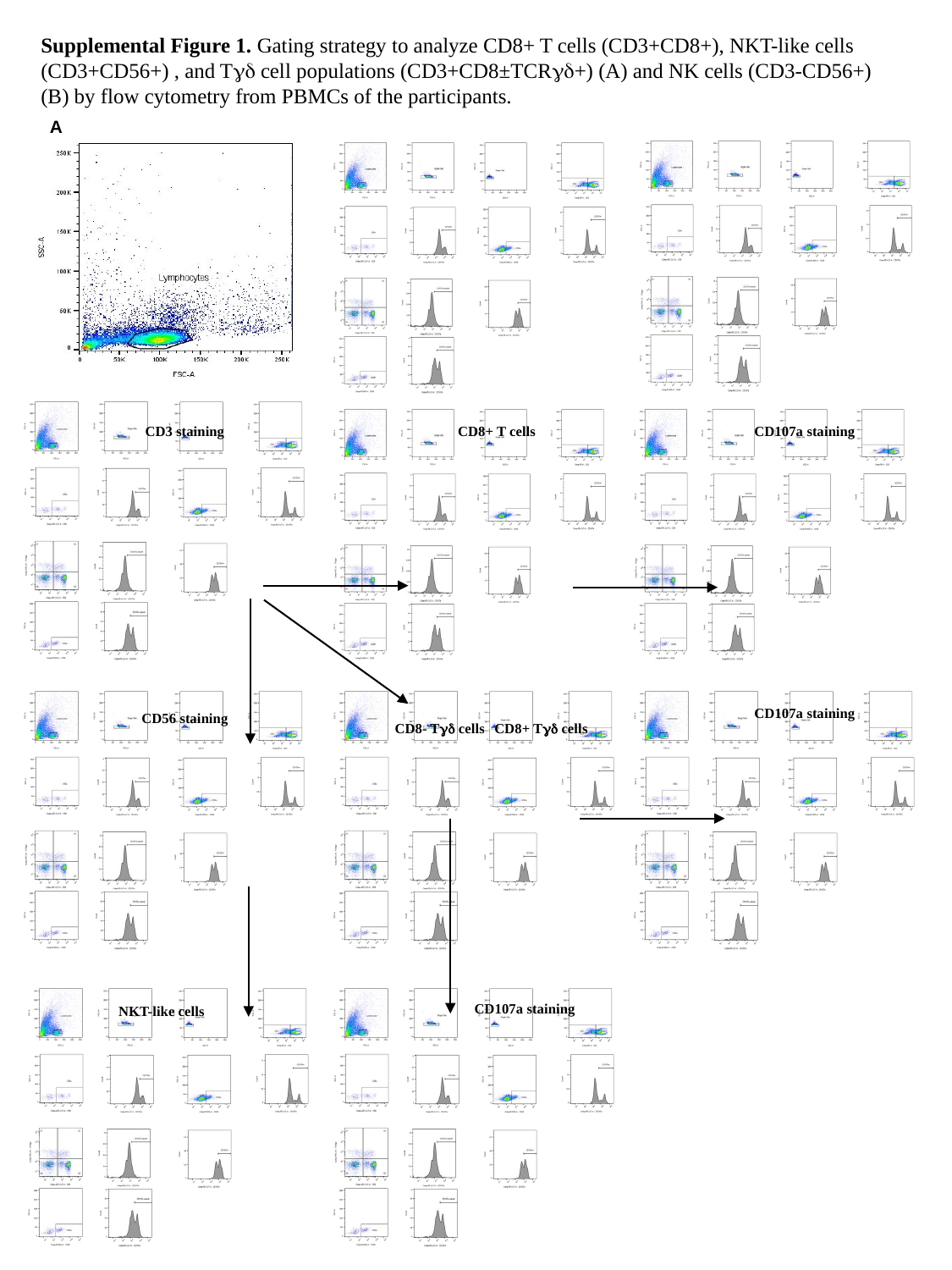

Supplemental Figure 1. Gating strategy to analyze CD8+ T cells (CD3+CD8+), NKT-like cells (CD3+CD56+) , and Tgd cell populations (CD3+CD8±TCRgd+) (A) and NK cells (CD3-CD56+) (B) by flow cytometry from PBMCs of the participants.
A
CD3 staining
CD8+ T cells
CD107a staining
CD107a staining
CD56 staining
CD8- Tgd cells
CD8+ Tgd cells
CD107a staining
NKT-like cells

## Slide 2
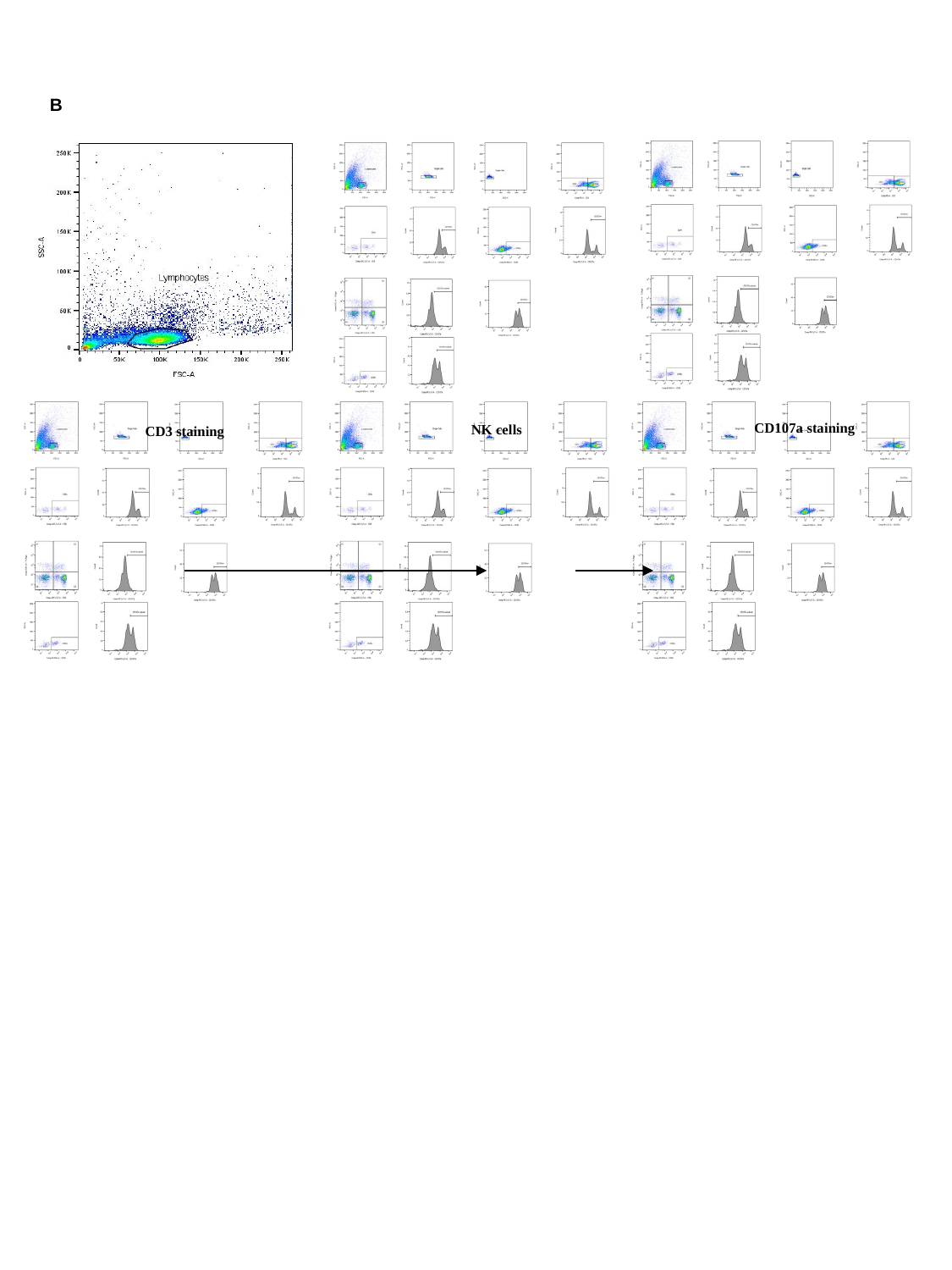

B
CD107a staining
NK cells
CD3 staining
