# Supplementary material for: Pre-existing cell populations with cytotoxic activity against SARS-CoV-2 in people with HIV and normal CD4/CD8 ratio previously unexposed to the virus
Source: Front Immunol. 2024 May 15;15:1362621. doi: 10.3389/fimmu.2024.1362621 (PMC11133563; doi:10.3389/fimmu.2024.1362621)
Supplement: Supplementary file 4 [file Presentation_2.pptx]

## Slide 1
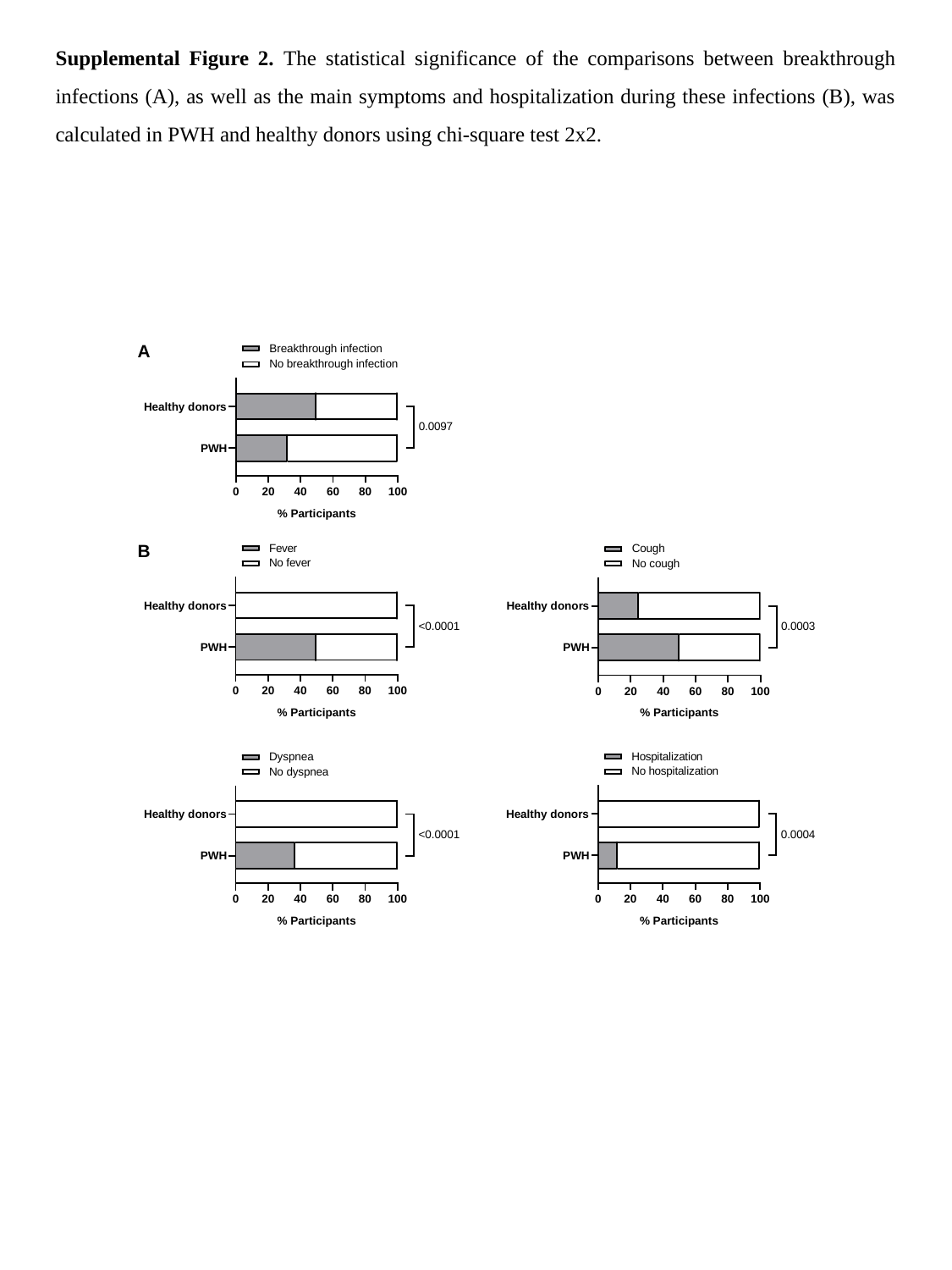

Supplemental Figure 2. The statistical significance of the comparisons between breakthrough infections (A), as well as the main symptoms and hospitalization during these infections (B), was calculated in PWH and healthy donors using chi-square test 2x2.
A
B
