# Supplementary material for: Pre-existing cell populations with cytotoxic activity against SARS-CoV-2 in people with HIV and normal CD4/CD8 ratio previously unexposed to the virus
Source: Front Immunol. 2024 May 15;15:1362621. doi: 10.3389/fimmu.2024.1362621 (PMC11133563; doi:10.3389/fimmu.2024.1362621)
Supplement: Supplementary file 5 [file Presentation_3.pptx]

## Slide 1
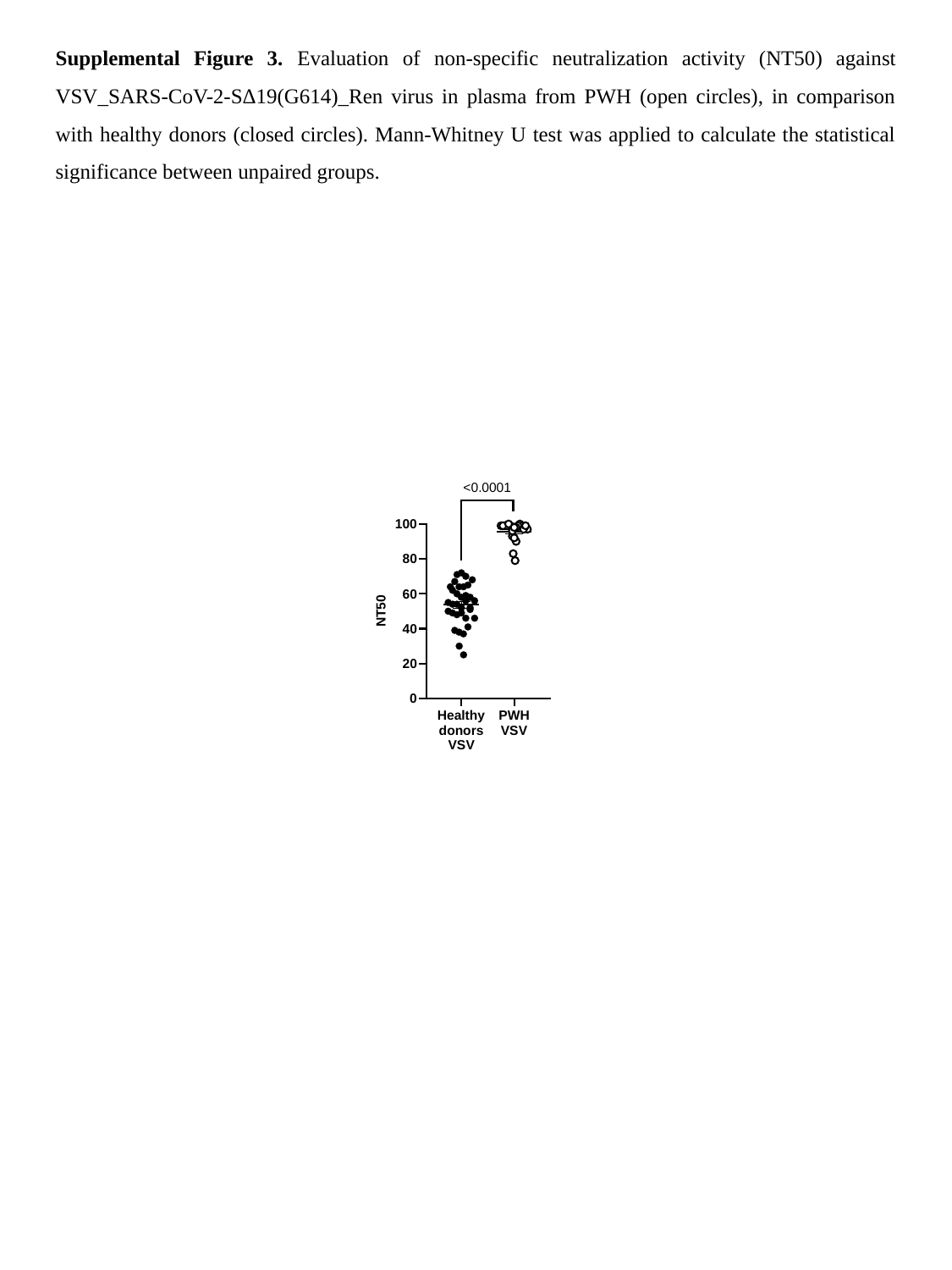

Supplemental Figure 3. Evaluation of non-specific neutralization activity (NT50) against VSV_SARS-CoV-2-SΔ19(G614)_Ren virus in plasma from PWH (open circles), in comparison with healthy donors (closed circles). Mann-Whitney U test was applied to calculate the statistical significance between unpaired groups.
