# Supplementary material for: Pre-existing cell populations with cytotoxic activity against SARS-CoV-2 in people with HIV and normal CD4/CD8 ratio previously unexposed to the virus
Source: Front Immunol. 2024 May 15;15:1362621. doi: 10.3389/fimmu.2024.1362621 (PMC11133563; doi:10.3389/fimmu.2024.1362621)
Supplement: Supplementary file 6 [file Presentation_4.pptx]

## Slide 1
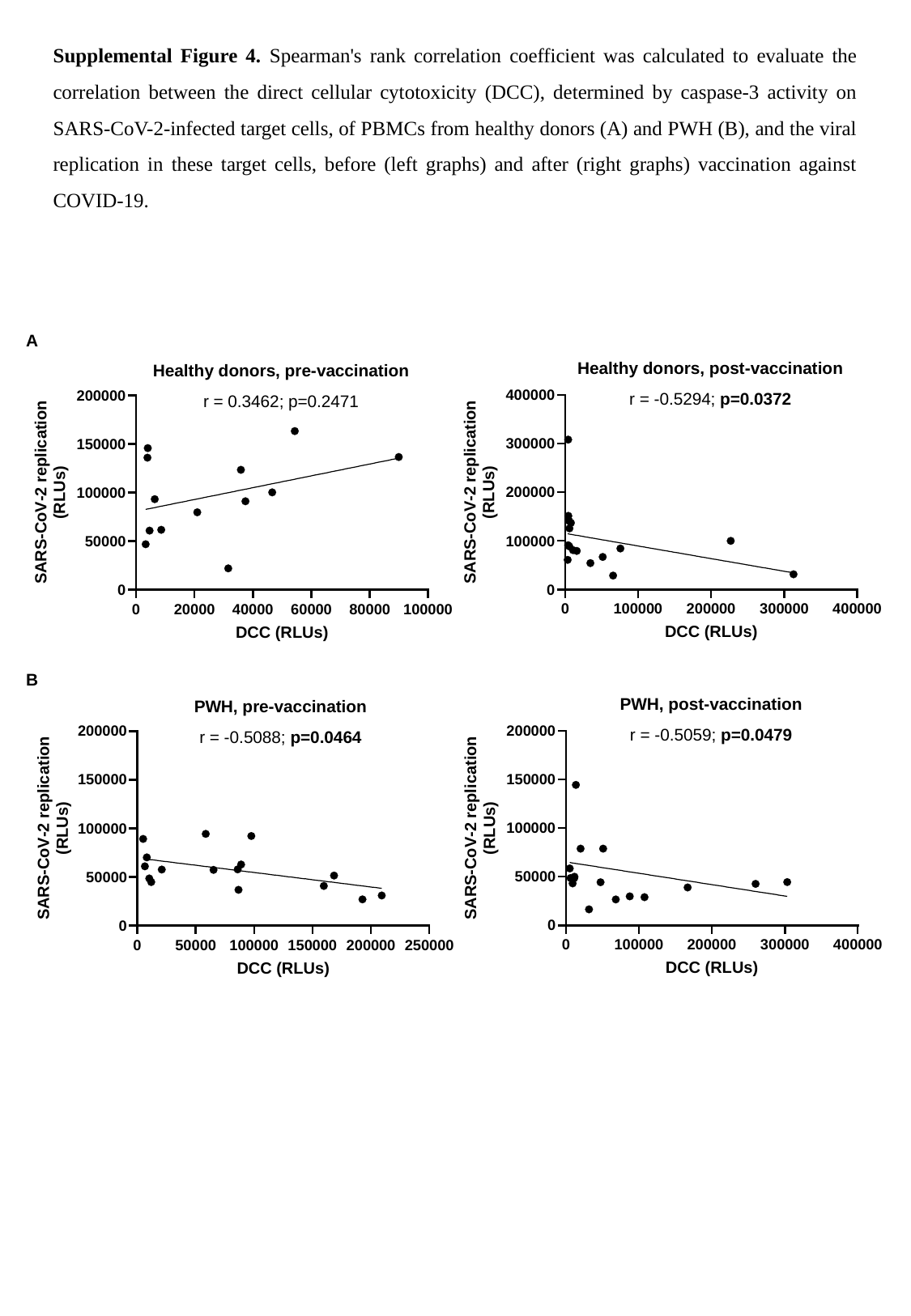

Supplemental Figure 4. Spearman's rank correlation coefficient was calculated to evaluate the correlation between the direct cellular cytotoxicity (DCC), determined by caspase-3 activity on SARS-CoV-2-infected target cells, of PBMCs from healthy donors (A) and PWH (B), and the viral replication in these target cells, before (left graphs) and after (right graphs) vaccination against COVID-19.
A
Healthy donors, post-vaccination
r = -0.5294; p=0.0372
Healthy donors, pre-vaccination
r = 0.3462; p=0.2471
B
PWH, post-vaccination
r = -0.5059; p=0.0479
PWH, pre-vaccination
r = -0.5088; p=0.0464
