# Supplementary material for: Pre-existing cell populations with cytotoxic activity against SARS-CoV-2 in people with HIV and normal CD4/CD8 ratio previously unexposed to the virus
Source: Front Immunol. 2024 May 15;15:1362621. doi: 10.3389/fimmu.2024.1362621 (PMC11133563; doi:10.3389/fimmu.2024.1362621)
Supplement: Supplementary file 7 [file Presentation_5.pptx]

## Slide 1
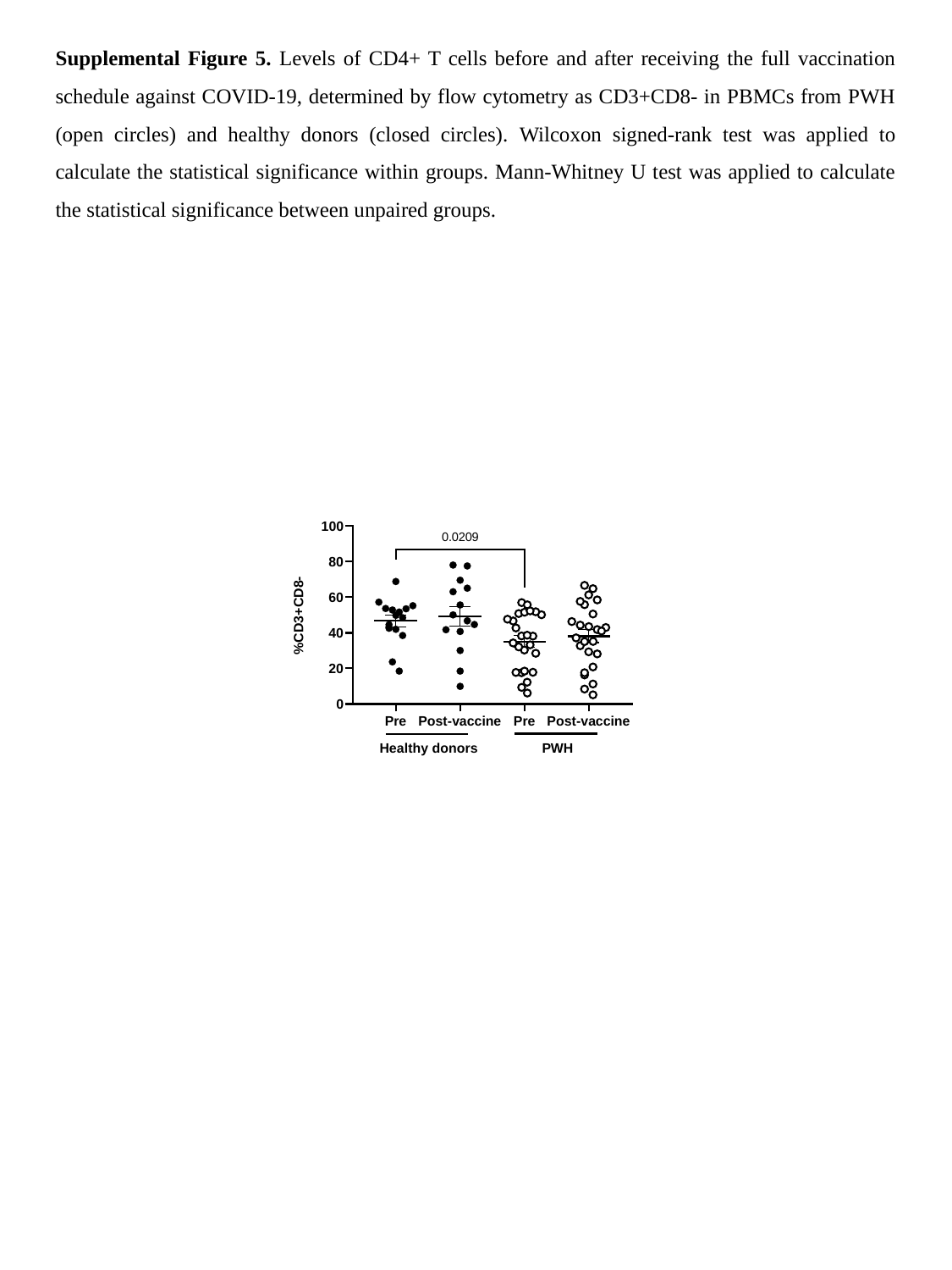

Supplemental Figure 5. Levels of CD4+ T cells before and after receiving the full vaccination schedule against COVID-19, determined by flow cytometry as CD3+CD8- in PBMCs from PWH (open circles) and healthy donors (closed circles). Wilcoxon signed-rank test was applied to calculate the statistical significance within groups. Mann-Whitney U test was applied to calculate the statistical significance between unpaired groups.
