# Supplementary material for: Pre-existing cell populations with cytotoxic activity against SARS-CoV-2 in people with HIV and normal CD4/CD8 ratio previously unexposed to the virus
Source: Front Immunol. 2024 May 15;15:1362621. doi: 10.3389/fimmu.2024.1362621 (PMC11133563; doi:10.3389/fimmu.2024.1362621)
Supplement: Supplementary file 8 [file Presentation_6.pptx]

## Slide 1
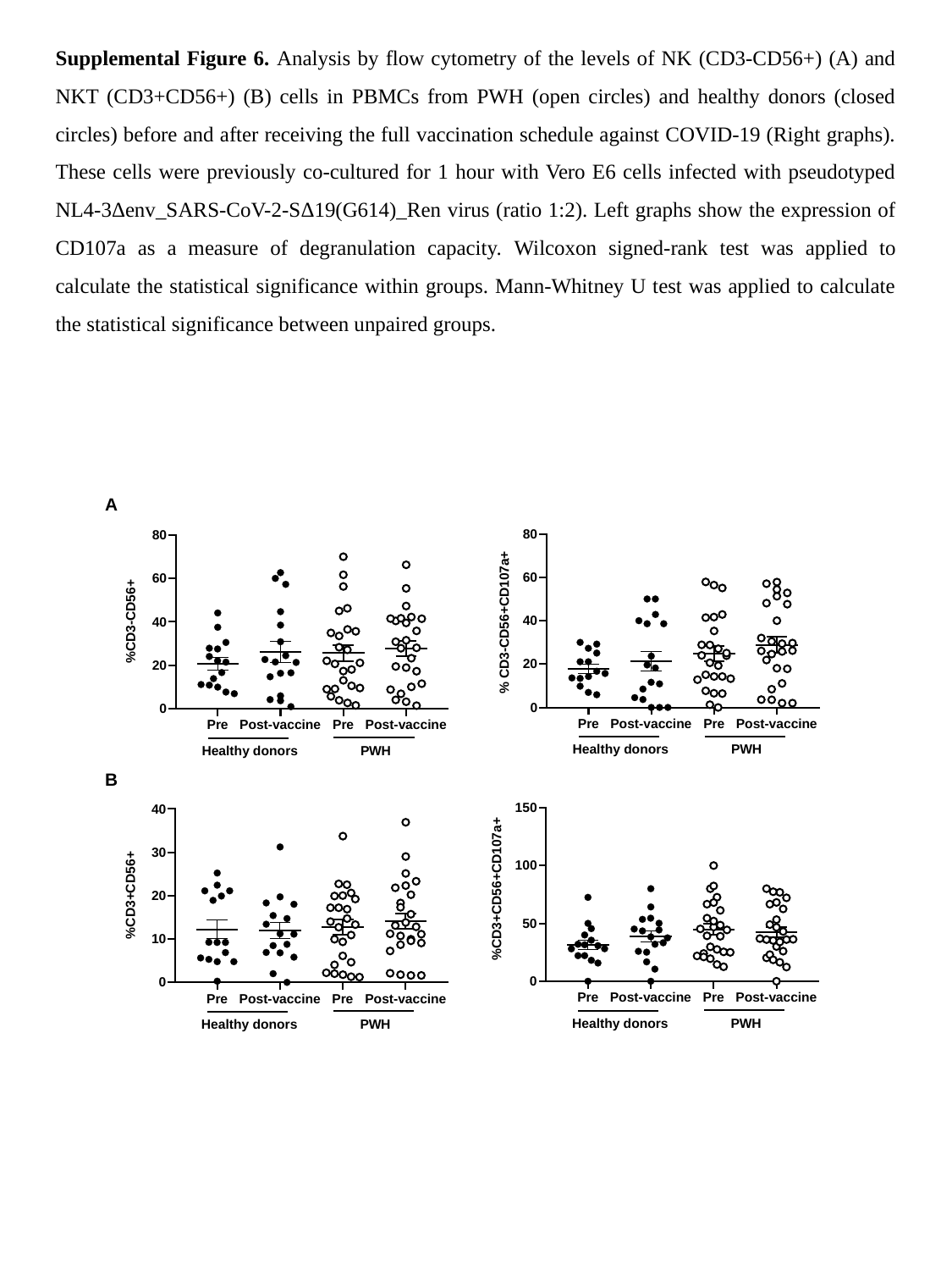

Supplemental Figure 6. Analysis by flow cytometry of the levels of NK (CD3-CD56+) (A) and NKT (CD3+CD56+) (B) cells in PBMCs from PWH (open circles) and healthy donors (closed circles) before and after receiving the full vaccination schedule against COVID-19 (Right graphs). These cells were previously co-cultured for 1 hour with Vero E6 cells infected with pseudotyped NL4-3Δenv_SARS-CoV-2-SΔ19(G614)_Ren virus (ratio 1:2). Left graphs show the expression of CD107a as a measure of degranulation capacity. Wilcoxon signed-rank test was applied to calculate the statistical significance within groups. Mann-Whitney U test was applied to calculate the statistical significance between unpaired groups.
A
B
