# Supplementary material for: Pre-existing cell populations with cytotoxic activity against SARS-CoV-2 in people with HIV and normal CD4/CD8 ratio previously unexposed to the virus
Source: Front Immunol. 2024 May 15;15:1362621. doi: 10.3389/fimmu.2024.1362621 (PMC11133563; doi:10.3389/fimmu.2024.1362621)
Supplement: Supplementary file 10 [file Presentation_8.pptx]

## Slide 1
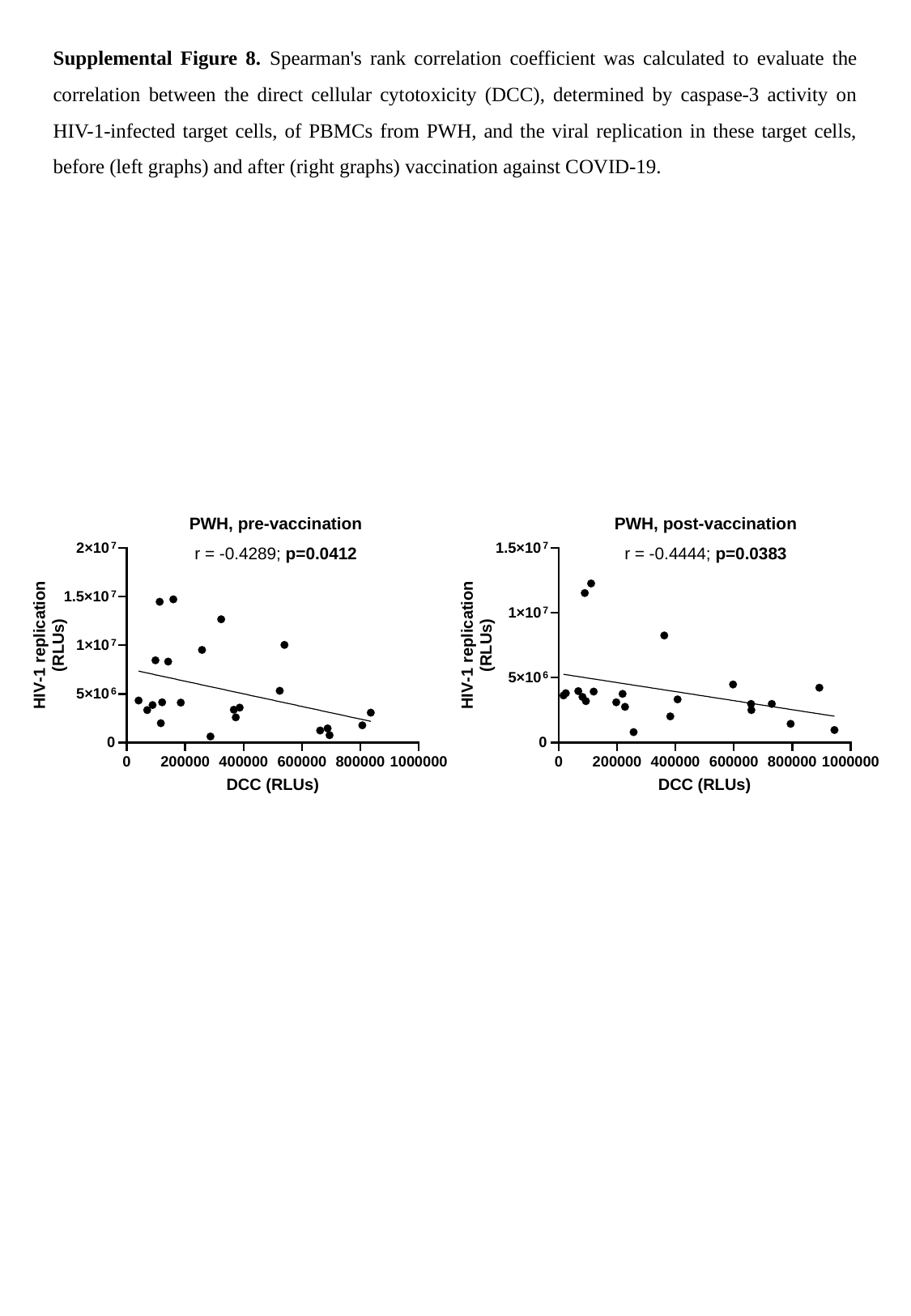

Supplemental Figure 8. Spearman's rank correlation coefficient was calculated to evaluate the correlation between the direct cellular cytotoxicity (DCC), determined by caspase-3 activity on HIV-1-infected target cells, of PBMCs from PWH, and the viral replication in these target cells, before (left graphs) and after (right graphs) vaccination against COVID-19.
PWH, pre-vaccination
r = -0.4289; p=0.0412
PWH, post-vaccination
r = -0.4444; p=0.0383
